# Assessment of fetal endometrial thickness: a key to the prenatal diagnosis of ovarian cysts

**DOI:** 10.1007/s00247-025-06494-x

**Published:** 2026-01-23

**Authors:** Minh-Huy Huynh, Laura De Leon Benedetti, Leny Mathew, Mohamed K Mohamed, Edward R Oliver, Suzanne E Debari, Juliana S Gebb, Nahla Khalek, Shelly Soni, Desiree Fiorentino, N. Scott Adzick, Beverly G Coleman

**Affiliations:** 1 Department of Radiology, Children’s Hospital of Philadelphia, 3401 Civic Center Blvd, Philadelphia, 19104 PA United States; 2 Children’s Hospital of Philadelphia, Philadelphia, United States; 3 Department of Radiology, University of Iowa Hospitals and Clinics, Iowa City, United States; 4 Department of Surgery, Children’s Hospital of Philadelphia, Philadelphia, United States

**Keywords:** Endometrium, Fetus, Magnetic resonance imaging, Ovarian cysts, Ultrasonography, prenatal

## Abstract

**Background:**

Fetal abdominopelvic cysts are relatively common, but distinguishing ovarian from non-ovarian cysts prenatally remains challenging because morphologic features overlap, and no imaging marker has been validated.

**Objective:**

To evaluate fetal endometrial thickness and cyst volume as imaging markers for the prenatal diagnosis of ovarian cysts.

**Materials and methods:**

We conducted a single-center, retrospective study of female fetuses with abdominopelvic cysts diagnosed on prenatal ultrasound and/or magnetic resonance imaging (MRI) between January 2010 and December 2024. Two blinded pediatric radiologists independently measured endometrial thickness and cyst volume, with discrepancies resolved by consensus. Postnatal confirmation of diagnosis was obtained through imaging, surgical pathology, or clinical follow-up. Statistical analyses included regression models adjusted for gestational age and receiver operating characteristic (ROC) analysis.

**Results:**

A total of 63 fetuses met inclusion criteria (40 ovarian cysts, 23 non-ovarian cysts) between 21 weeks and 40 weeks of gestation. Fetuses with ovarian cysts underwent ultrasound at later gestational ages than those with non-ovarian cysts (median, 35.0 weeks vs. 27.9 weeks; *P*<0.001), and MRI showed a similar difference (median, 34.4 weeks vs. 27.9 weeks; *P*<0.001). Ovarian cysts were associated with significantly greater endometrial thickness on ultrasound (median 3.2 mm vs. 1.3 mm, *P*<0.001) and MRI (2.2 mm vs. 1.2 mm, *P*<0.001). Cyst volumes were larger in ovarian cysts (median, 45.4 mL vs. 2.8 mL; *P*<0.001), although volume was not independently associated with ovarian cyst diagnosis after adjustment for gestational age (*P*=0.36). Endometrial thickness remained independently associated with ovarian cysts after adjustment for gestational age (ultrasound coefficient, 1.56 [95% CI, 0.84–2.26]; MRI coefficient, 0.81 [95% CI, 0.41–1.21]). ROC analysis demonstrated excellent diagnostic performance, with an ultrasound threshold of 1.9 mm yielding 100% sensitivity and specificity (AUC, 1.00 [95% CI, 1.00–1.00]). In contrast, cyst volume showed only moderate discriminatory ability (AUC, 0.82 [95% CI, 0.71–0.93]).

**Conclusion:**

Fetal endometrial thickness is a robust imaging marker for the prenatal diagnosis of ovarian cysts. Incorporating this parameter into routine prenatal imaging may enhance diagnostic accuracy, guide counseling, and improve perinatal management.

**Graphical abstract:**

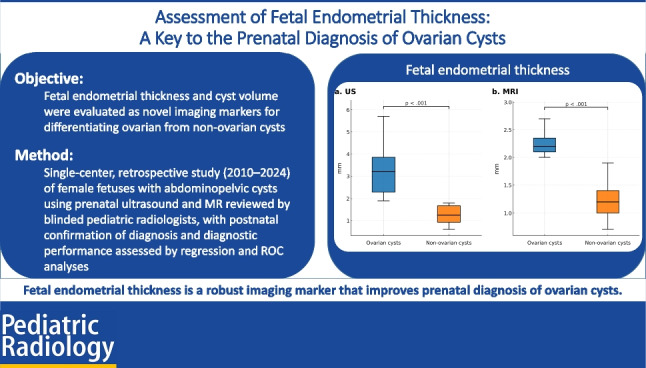

## Introduction

Fetal abdominopelvic cysts are relatively common incidental findings on second- and third-trimester ultrasound, with an estimated incidence of approximately 1 in 1,000 pregnancies [1, 2]. These cysts typically appear as round, anechoic lesions and encompass a broad differential diagnosis that includes adnexal, gastrointestinal, renal, lymphatic, and neoplastic etiologies [3–5]. Because imaging features often overlap, accurate lesion localization and confirmation of fetal gender are critical for narrowing the differential diagnosis.

Among these entities, fetal ovarian cysts are the most frequently encountered abdominopelvic cysts in female fetuses, with an estimated prevalence of 1 in 2,500 live female births [3, 6–8]. They usually arise in the third trimester due to maternal and placental estrogen and gonadotropin stimulation of the fetal ovary [3–6]. While many are physiologic and resolve spontaneously, complications such as torsion, hemorrhage, rupture, and ovarian loss are well documented [6, 9–11]. These adverse outcomes may necessitate surgical intervention and can jeopardize future fertility, highlighting the importance of timely and accurate prenatal diagnosis to guide prognosis, monitoring, and perinatal management.

Accurate prenatal identification of ovarian cysts remains challenging, as morphologic features alone may be misinterpreted or indistinguishable from non-ovarian cysts. Although the imaging characteristics of fetal ovarian cysts have been described extensively [4, 5], no physiologic imaging marker has been established to improve specificity. Fetal endometrial thickness represents a promising candidate. In postnatal populations, endometrial thickness is a well-validated, estrogen-responsive marker for assessing hormonal status, pubertal development, and abnormal uterine bleeding [12–14]. Estrogen receptors are present in the fetal endometrium by the late second and third trimesters, and histologic studies confirm its responsiveness to transplacental estrogen exposure [15]. Thus, prenatal endometrial thickening may serve as a noninvasive, physiologic marker of intrauterine estrogen stimulation, reflecting the influence of maternal and placental hormones on ovarian cyst formation and thereby improving diagnostic confidence.

Cyst volume is another underexplored diagnostic parameter. Most studies only report the maximum cyst diameter, which does not fully capture the burden of disease. Volume provides a three-dimensional assessment that may better reflect both hormonal stimulation and mass effect. Larger cysts are more likely to undergo torsion or require surgical intervention [6–9]; however, cyst volume has not been systematically evaluated as a discriminator between ovarian and non-ovarian cysts.

In this study, we propose fetal endometrial thickness and cyst volume as novel sonographic markers to improve the prenatal diagnosis of fetal ovarian cysts. We hypothesized that fetuses with ovarian cysts would demonstrate significantly greater endometrial thickness and cyst volumes compared with non-ovarian cysts, and that incorporating these markers would enhance diagnostic confidence.

## Materials and methods

This single-center, retrospective study was approved by the Institutional Review Board (IRB) of our tertiary care children’s hospital. Female fetuses with abdominopelvic cysts detected on prenatal imaging between January 2010 and December 2024 were eligible for inclusion.

Inclusion criteria were as follows: (1) detection of an abdominopelvic cyst on prenatal ultrasound, with magnetic resonance imaging (MRI) included when available; (2) adequate prenatal imaging permitting assessment of the uterus and endometrium by ultrasound and/or MRI when available; and (3) postnatal confirmation of diagnosis through imaging, surgical pathology, or clinical follow-up. Exclusion criteria were as follows: (1) known chromosomal abnormalities, (2) major congenital anomalies, (3) suboptimal imaging quality precluding measurement, and (4) loss to follow-up without postnatal confirmation. Cases meeting inclusion criteria were categorized into two diagnostic groups: ovarian cysts and non-ovarian cysts.

### Data collection

Demographic and clinical data were extracted from the electronic medical records. Variables collected included maternal age, maternal ethnicity, gestational age at imaging, cyst type identified on prenatal imaging, postnatal diagnosis and clinical outcomes, and availability of surgical pathology.

Ovarian cysts were further classified as simple or complicated. Complicated cysts were defined by the presence of hemorrhage or imaging features suggestive of ovarian torsion. For non-ovarian cysts, the suspected diagnosis (e.g., enteric duplication, lymphatic malformation) was recorded from the interpreting radiologist’s report. Clinical outcomes were categorized as spontaneous resolution or surgical intervention. In cases requiring surgery, operative and pathology reports were reviewed when available.

### Imaging analysis

Fetal endometrial thickness and cyst volume were assessed on prenatal ultrasound and magnetic resonance imaging (MRI).

Endometrial thickness was measured independently by two pediatric radiologists, one with 3 years of dedicated fetal imaging experience and the other with more than 20 years of experience, both blinded to the postnatal diagnosis. When measurements differed, the images were reviewed jointly and a consensus measurement was recorded for analysis, typically aligning with the assessment of the more senior radiologist. No formal interobserver reproducibility assessment was performed.

### Ultrasound technique

Ultrasound examinations were performed using curved C9-2 and linear eL18-4 transducers. Optimal visualization of the uterus was achieved when the fetal spine was positioned superiorly, allowing the rectosigmoid colon to be seen posteriorly, the uterus centrally, and the bladder anteriorly. Although sagittal uterine views are not part of our institution’s standard fetal ultrasound protocol, some members of the fetal imaging division began routinely obtaining sagittal views of the fetal uterus in female fetuses with abdominopelvic cysts during the study period. This practice was not uniformly adopted at the outset and became more consistent later as growing interest in endometrial assessment developed.

**Fig. 1:**
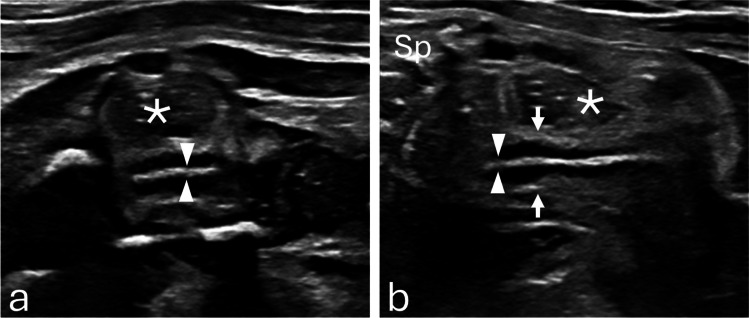
**a** Transverse and (**b**) sagittal grayscale ultrasound images at 29 weeks 4 days show the uterus (arrows) and non-thickened endometrium (arrowheads). The rectum (asterisk) lies posterior to the uterus. The fetal spine (Sp) is oriented anteriorly for image optimization. Thickness was measured as the maximal anteroposterior dimension of the echogenic endometrium, outer-to-outer between the anterior and posterior basal echogenic borders

Endometrial thickness was measured in a midline sagittal view of the uterus, typically obtained near the fundus or upper body. The endometrium was identified by its layered echotexture: a thin echogenic line in the early proliferative phase; a multilayered appearance in the late proliferative phase, consisting of basal echogenic lines and intervening hypoechoic functional layers; and a uniformly echogenic band in the secretory phase. Thickness was measured as the maximum anteroposterior dimension of the echogenic endometrium, placed outer-to-outer from the anterior to the posterior basal echogenic borders, oriented perpendicular to the uterine axis. Adjacent hypoechoic myometrium and intrauterine fluid were excluded (Fig. 1).

### MRI technique

**Fig. 2:**
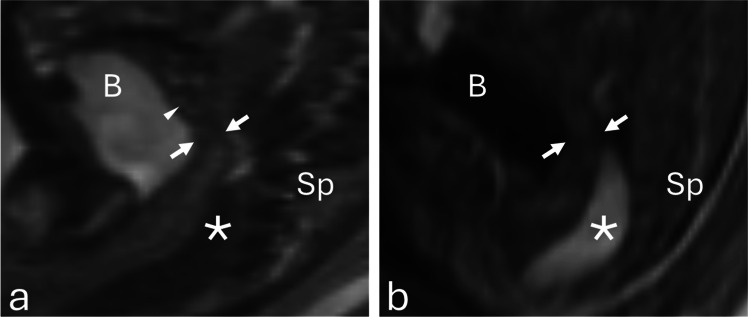
**a** Sagittal T2-weighted MR image shows the uterus posterior to the urinary bladder (B), with hypointense myometrium (arrows) and mildly hyperintense endometrium (arrowhead). **b** Sagittal T1-weighted MR image shows T1-hyperintense meconium within the rectosigmoid colon (asterisk), posterior to the uterus (arrows). Measurements were made from the outer margin to the outer margin across the anteroposterior dimension of the endometrium

MRI was performed on a 1.5-T system using T2-weighted HASTE sequences with 3-mm slice thickness. Endometrial thickness was measured in the midsagittal plane, where the endometrium appears as a thin, relatively hyperintense linear zone compared with the surrounding hypointense myometrium. Measurements were made from the outer margin to the outer margin across the anteroposterior dimension of the endometrium (Fig. 2).

Cyst volume was calculated on ultrasound and MR using the standard ellipsoid formula: length×width×height×0.52.

### Statistical analysis

Continuous variables were summarized as medians with interquartile ranges (IQR) and categorical variables as counts and percentages. Between-group comparisons were made using Wilcoxon rank-sum or chi-square tests, as appropriate. Linear regression models adjusted for gestational age were used to assess independent associations of cyst type with endometrial thickness and volume. Receiver operating characteristic (ROC) curve analysis was used to determine diagnostic thresholds, sensitivity, specificity, and predictive values. Statistical significance was defined as *P*<0.05. Analyses were performed using R (version 4.2.0).

## Results

The final study population consisted of 63 female fetuses with abdominal or pelvic cysts. All included cases had at least one prenatal imaging modality—ultrasound or MRI—sufficient for endometrial thickness measurement. The median maternal age was 36 years (range, 25–50 years), and most patients were White (61.9%). The median gestational age at the diagnostic ultrasound was 34.0 weeks (range, 20.5–40.3 weeks). Endometrial thickness could not be assessed on ultrasound in 17 fetuses (27.0%) because sagittal uterine views were unavailable or the endometrium could not be adequately measured, as this imaging view was not part of the standard protocol during the study period. Missing ultrasound measurements occurred in nine non-ovarian cyst cases (39.1%) and eight ovarian cyst cases (20.0%). MRI endometrial thickness measurements were missing in 44 fetuses (69.8%); these included 11 non-ovarian cyst cases (47.8%) and 33 ovarian cyst cases (82.5%) (Table 1).

**Table 1:** Patient demographics (*N*=63)

Characteristic	Value
Maternal age, years	Median 36 (25–50)
Maternal ethnicity	White: 39 (61.9%) Black: 9 (14.3%) Asian: 7 (11.1%) Other: 8 (12.7%)
Gestational age at ultrasound, weeks	Median 34.0 (20.5–40.3)
Gestational age at MRI, weeks	Median 32.8 (22.6–37.6)

**Fig. 3:**
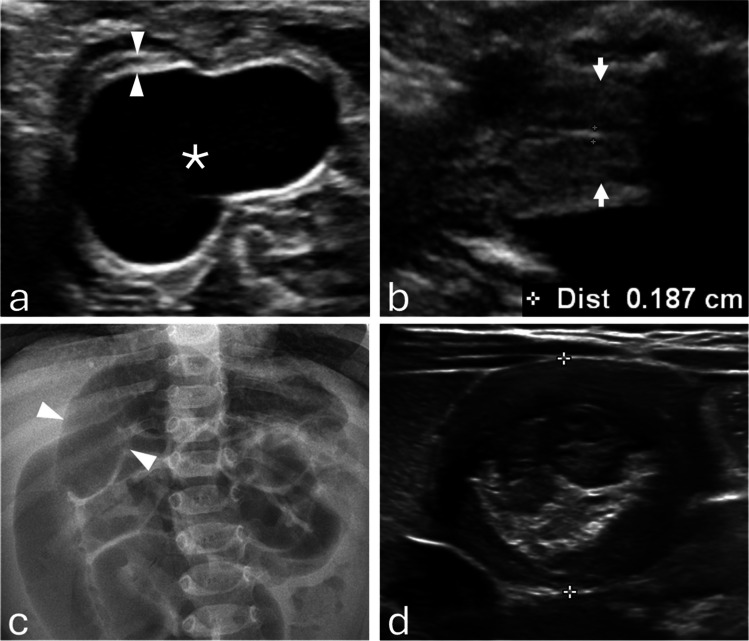
**a** Prenatal grayscale ultrasound at 34 weeks 3 days demonstrates a pathology-proven enteric duplication cyst (asterisk) with the classic gut signature, consisting of an echogenic inner mucosal layer and a hypoechoic outer muscular layer (arrowheads). **b** Sagittal grayscale ultrasound shows the fetal uterus (arrows) with an endometrium measuring < 2 mm. **c** At 4 months of age, frontal abdominal radiograph demonstrates small-bowel obstruction (arrowheads). **d** Grayscale ultrasound demonstrates an ileocolic intussusception caused by the enteric duplication cyst. The infant underwent surgical resection

Of 63 cases, 40 (63.5%) were ovarian cysts (25 hemorrhagic, 15 simple) and 23 (36.5%) were non-ovarian cysts, most often enteric duplication (12 of 23 [52.2%]) (Fig. 3). Ovarian torsion occurred in 20 of 40 ovarian cysts (50.0%), including 17 of 25 hemorrhagic cysts (68.0%) and 3 of 15 simple cysts (20.0%). Torsion was significantly more frequent in hemorrhagic than in simple cysts (OR, 0.12 [95% CI, 0.02–0.61]; *P*=0.008) (Fig. 6). Torsion was confirmed intraoperatively and corroborated by pathology (Table 2).

**Table 2:** Clinical characteristics (*N*=63)

Characteristic	Value	*P*-value/OR
Diagnosis	Ovarian cysts (40/63, 63.5%) – Simple: 15 (37.5%) – Hemorrhagic: 25 (62.5%) Non-ovarian cysts (23/63, 36.5%) – Enteric duplication: 12 (52.2%) – Lymphatic malformation: 1 (4.3%) – Choledochal: 2 (8.7%) – Meconium pseudocyst: 3 (13.0%) – Hepatic: 2 (8.7%) – Renal: 1 (4.3%) – Teratoma: 1 (4.3%) – Hematocolpos: 1 (4.3%)	–
Ovarian torsion^a^	Yes: 20 (50.0%) No: 20 (50.0%)	Hemorrhagic vs. simple: OR=0.12, *P*=0.008
Clinical outcomes	Ovarian cysts: 11 resolved spontaneously (27.5%), 22 required surgery (55.0%) non-ovarian cysts: 5 resolved spontaneously (21.7%), 18 required surgery (78.3%)	Ovarian vs. non-ovarian surgery: OR=0.34, *P*=0.10
Pathology available	Yes: 37 (58.7%) No: 26 (41.3%)	–

Data shown as *n* (%) unless otherwise indicated.

^a^*Among ovarian cyst cases only.

^b^Percentages for ovarian and non-ovarian subtypes are calculated from within-group totals; overall

percentages for ovarian vs. non-ovarian cysts are based on the total cohort (*N* = 63).

**Fig. 4:**
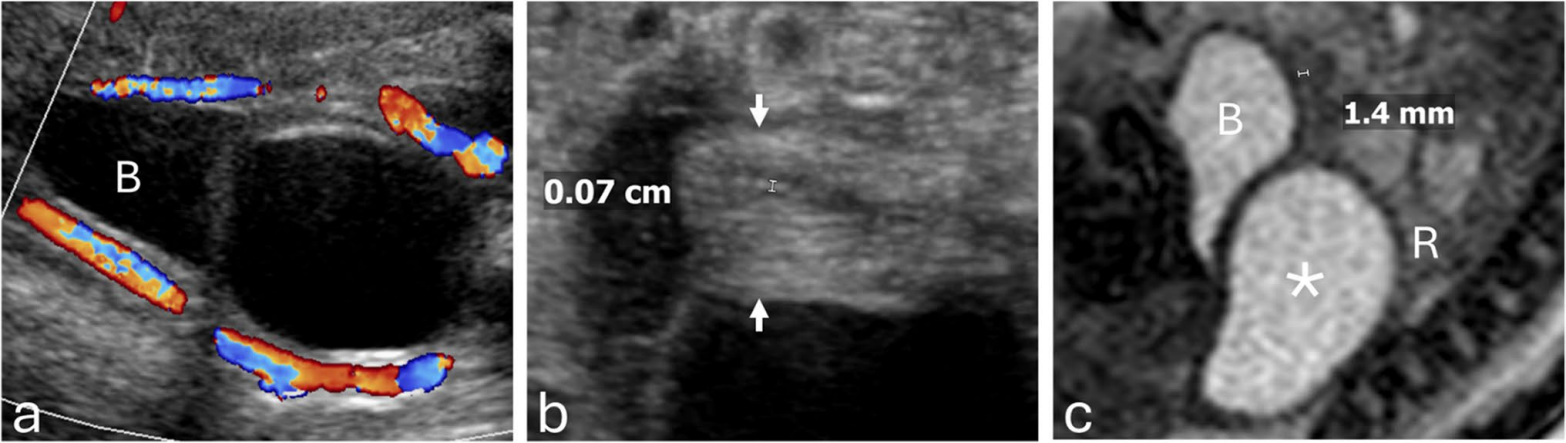
**a** Prenatal grayscale ultrasound with color Doppler at 33 weeks shows imperforate hymen with hydrocolpos. The fluid-distended vagina (asterisk) appears cystic, posterior and slightly inferior to the bladder (B) and anterior to the rectosigmoid colon (R). **b** Sagittal grayscale ultrasound shows the uterus (arrows) with a non-thickened endometrium measuring < 2 mm. **c** Sagittal T2-weighted MR image confirms hydrocolpos (asterisk) and a non-thickened endometrium. The infant underwent postnatal hymenectomy

**Fig. 5:**
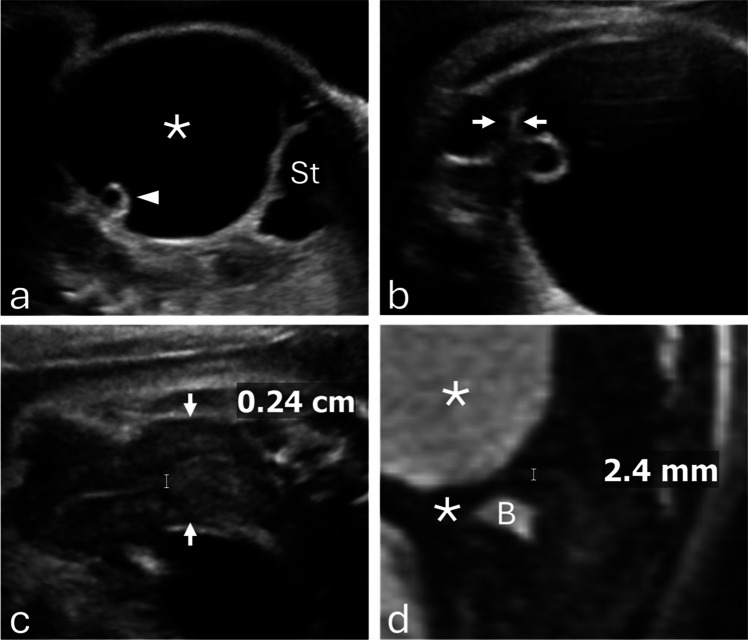
**a** Prenatal sagittal grayscale ultrasound at 36 weeks 5 days demonstrates a large unilocular ovarian cyst (asterisk) extending from the left pelvis to the upper abdomen adjacent to the stomach, containing a “daughter cyst” (arrowhead), characteristic of ovarian cyst. **b** A close-up image demonstrates peripheral displacement of the ovarian stroma (arrows).** c** Sagittal grayscale ultrasound shows the fetal uterus (arrows) with a thickened endometrium measuring > 2 mm. **d** Sagittal T2-weighted MR image confirms a thickened endometrium. The infant underwent exploratory laparotomy with cyst drainage, which confirmed a simple ovarian cyst

**Fig. 6:**
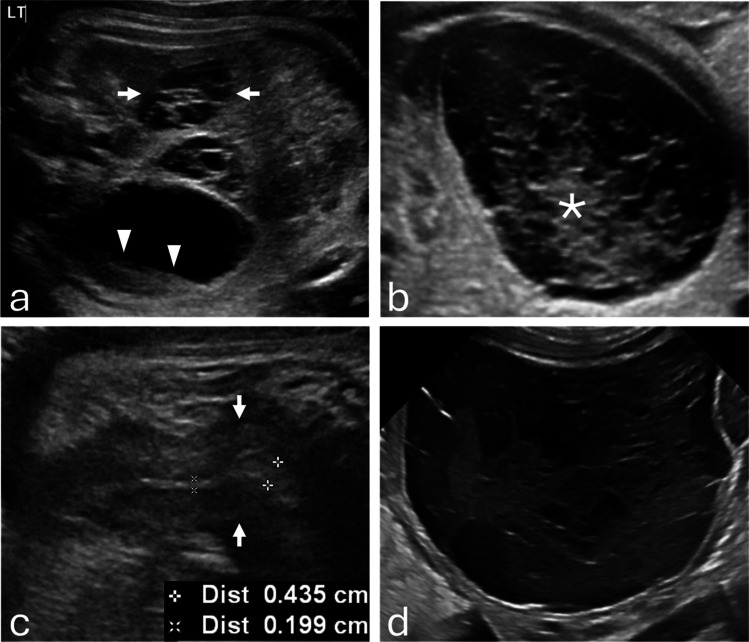
**a** Prenatal grayscale ultrasound at 30 weeks demonstrates a hormonally stimulated right ovary containing a hemorrhagic cyst with a fluid–debris level (arrowheads) and preserved stroma with follicles. The left ovary is also hormonally stimulated with multiple follicles (arrows). **b** At 36 weeks, the cyst evolved to a reticular pattern with echogenic, non-shadowing retractile clot (asterisk). **c** Sagittal grayscale ultrasound shows the fetal uterus (arrows) with a thickened endometrium at the fundus measuring > 2 mm. **d** Postnatal grayscale ultrasound at day 4 demonstrates a persistent hemorrhagic ovarian cyst measuring 74 mL in volume. The infant underwent surgery, which confirmed a necrotic ovarian cyst with hemorrhage and calcifications due to prenatal torsion

Spontaneous resolution, defined as absence of cyst visualization on the latest follow-up imaging, occurred in 11 ovarian cysts (27.5%) and five non-ovarian cysts (21.7%). Surgery was required in 18 non-ovarian cysts (78.3%) (Figs. 3, 4) and 22 ovarian cysts (55.0%) (Figs. 5, 6); the difference between groups was not statistically significant (OR, 0.34 [95% CI, 0.10–1.14]; *P*=0.10). Pathology reports were available for 37 of 63 cases (58.7%), including all surgically resected ovarian cysts. Among non-ovarian cysts, pathology was obtained in nearly all surgical cases; however, in one case, no specimen was obtained, and the diagnosis was confirmed intraoperatively.

### Gestational age and imaging timing

**Fig. 7:**
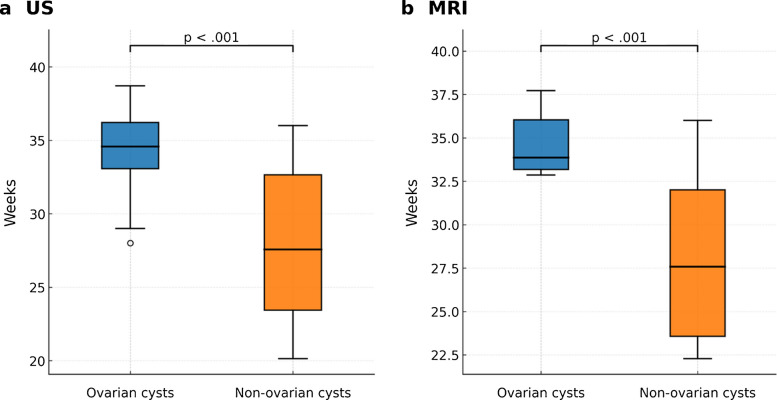
Gestational age and imaging timing. **a** Ultrasound examinations were performed at later gestational ages in fetuses with ovarian cysts compared with those with non-ovarian cysts (median 35.0 weeks [IQR 33.8–36.1] vs. 27.9 weeks [IQR 23.8–33.1]; *P* < 0.001). **b** Among the 19 fetuses who also underwent MRI (30.2%), gestational age at the time of imaging was higher in the ovarian cyst group (median 34.4 weeks [IQR 34.0–34.8]) than in the non-ovarian group (median 27.9 weeks [IQR 23.9–32.8]; *P* < 0.001)

Fetuses with ovarian cysts underwent ultrasound at significantly later gestational ages than those with non-ovarian cysts (median 35.0 weeks [IQR, 33.8–36.1] vs. 27.9 weeks [IQR, 23.8–33.1]; *P*<0.001). MRI was performed in 19 cases (30.2%) at a median of 34.4 weeks (IQR, 34.0–34.8) for the ovarian cyst group and 27.9 weeks (IQR, 23.9–32.8) for the non-ovarian cyst group (*P*<0.001) (Fig. 7).

(Table 3).

**Table 3:** Imaging parameters by diagnosis

Variable	Non-ovarian cyst (*n*=23)	Ovarian cyst (*n*=40)	Overall (*N*=63)	*P*-value
Gestational age at US, weeks	27.9 (23.8–33.1)	35.0 (33.8–36.1)	34.0 (31.0–35.4)	<0.001
Gestational age at MRI, weeks	27.9 (23.9–32.8)	34.4 (34.0–34.8)	32.8 (26.4–34.4)	<0.001
Cyst volume, mL (US)	2.8 (0.8–7.4)	45.4 (25.7–79.4)	28.0 (4.1–63.9)	<0.001
Endometrial thickness, mm (US)	1.3 (0.9–1.7)	3.2 (2.3–3.9)	2.35 (1.7–3.6)	<0.001
Endometrial thickness, mm (MRI)	1.2 (1.0–1.4)	2.2 (2.1–2.4)	1.4 (1.2–2.1)	<0.001

Values are median (IQR). Missing data: gestational age at MRI 44 (69.8%), endometrial thickness by ultrasound 17 (27.0%), endometrial thickness by MRI 44 (69.8%). US, ultrasound.

### Endometrial thickness

Endometrial thickness on ultrasound was measurable in 46 of 63 cases (73.0%). On ultrasound, fetuses with ovarian cysts had significantly greater endometrial thickness compared with non-ovarian cysts (median, 3.2 mm [IQR, 2.3–3.9] vs. 1.3 mm [IQR, 0.9–1.7]; *P*<0.001).

**Fig. 8:**
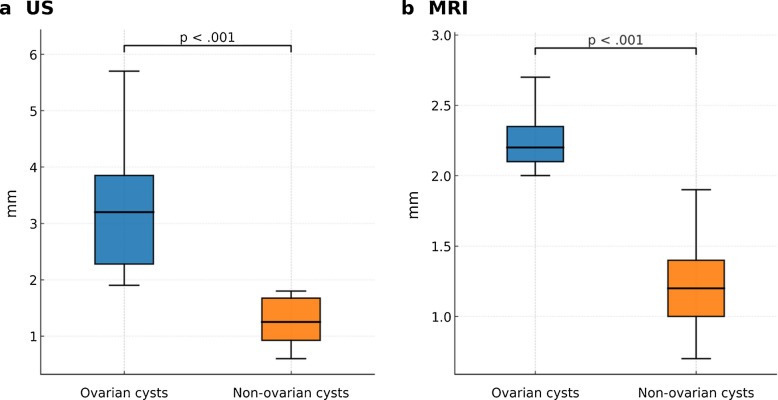
Endometrial thickness. **a** On ultrasound, fetuses with ovarian cysts demonstrated greater endometrial thickness compared with fetuses with non-ovarian cysts (median 3.2 mm [IQR 2.28–3.85] vs. 1.25 mm [IQR 0.93–1.68]; *P* < 0.001). This difference remained significant after adjusting for gestational age (coefficient 1.56; 95% CI 0.84–2.26; *P* < 0.001).** b** In the subset of 19 fetuses evaluated with MRI, endometrial thickness was again greater in the ovarian cyst group (median 2.2 mm [IQR 2.1–2.35]) compared with the non-ovarian group (median 1.2 mm [IQR 1.0–1.4]; *P* < 0.001), with significance persisting after adjustment for gestational age (coefficient 0.81; 95% CI 0.41–1.21; *P* < 0.001)

Among the 19 cases with MRI, endometrial thickness was also greater in ovarian cysts (median 2.2 mm [IQR, 2.1–2.4]) than in non-ovarian cysts (1.2 mm [IQR, 1.0–1.4]; *P*<0.001) (Fig. 8).

### Cyst volume

**Fig. 9:**
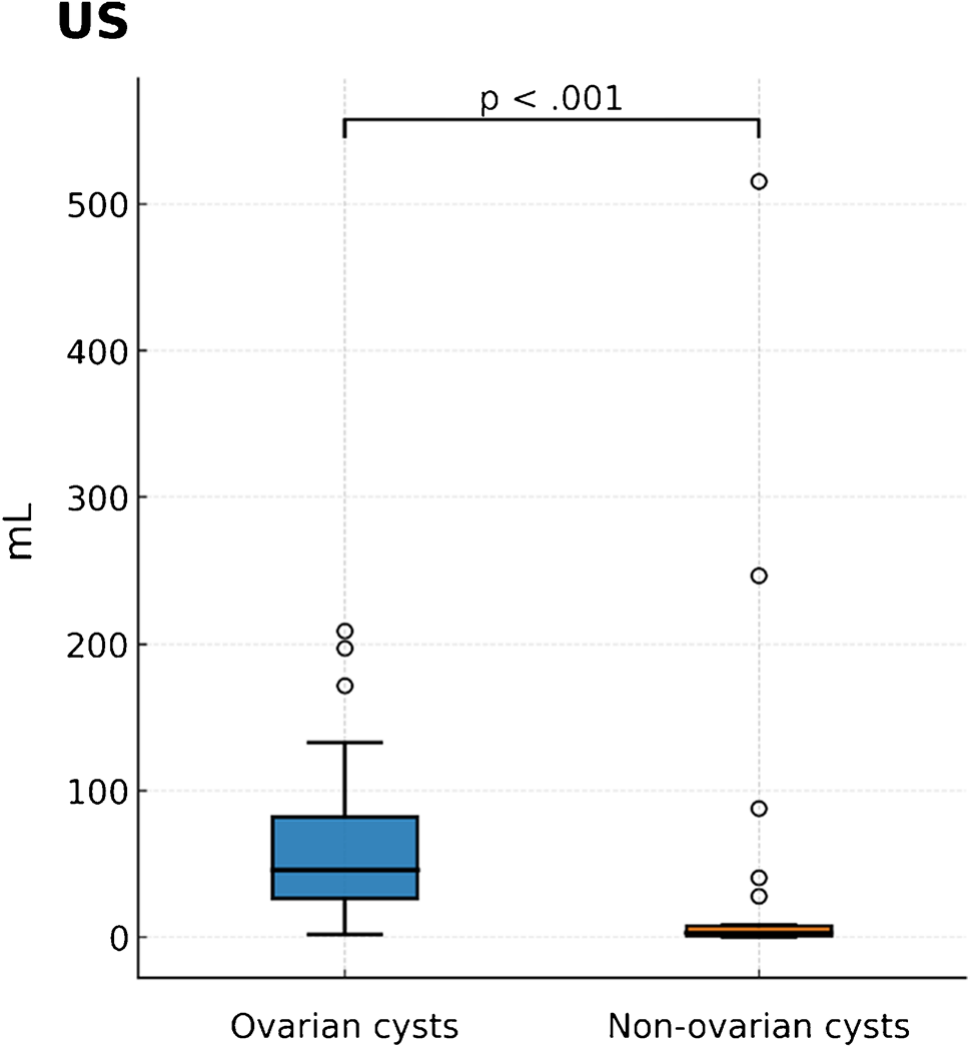
Cyst volume on ultrasound. Median cyst volume was substantially larger in ovarian cysts than in non-ovarian cysts (45.4 mL [IQR 25.7–79.4] vs. 2.8 mL [IQR 0.76–7.41]; *P*<0.001). After accounting for gestational age, cyst volume was not independently predictive of ovarian cyst diagnosis (coefficient −24.05; 95% CI −76.45 to 28.36; *P*=0.36)

Cyst volumes were significantly larger in ovarian cysts (median 45.4 mL [IQR, 25.7–79.4]) compared with non-ovarian cysts (2.8 mL [IQR, 0.8–7.4]; *P*<0.001). However, after adjusting for gestational age, cyst volume was not independently associated with ovarian cyst diagnosis (coefficient, −24.05 [95% CI, −76.45 to 28.36]; *P*=0.36) (Fig. 9) (Table 4).

**Table 4:** Diagnostic performance of imaging markers for ovarian cyst diagnosis

Parameter	Modality	Threshold	Sensitivity, %	Specificity, %	PPV, %	NPV, %	AUC (95% CI)
Endometrial thickness	Ultrasound	1.9 mm	100	100	100	100	1.00 (1.00–1.00)
Endometrial thickness	MRI	1.8 mm^a^	100	91.7	87.5	100	0.99 (0.96–1.00)
Cyst volume	Ultrasound	11.9 mL	90.0	78.3	87.7	81.6	0.82 (0.71–0.93)

*PPV*, positive predictive value; *NPV*, negative predictive value; *AUC*, area under the ROC curve; *CI*, confidence interval.^a^Threshold by Youden index

Multivariable linear regression models were used to evaluate whether endometrial thickness remained independently associated with ovarian cyst diagnosis after adjusting for gestational age. On ultrasound, ovarian cyst diagnosis was independently associated with greater endometrial thickness (coefficient, 1.56 [95% CI, 0.84–2.26]; *P*<0.001). Gestational age was also positively associated with endometrial thickness (coefficient, 0.08 mm per week [95% CI, 0.04–0.12]; *P*<0.001).

On MRI, ovarian cysts similarly showed increased endometrial thickness (coefficient, 0.81 [95% CI, 0.41–1.21]; *P*<0.001), and gestational age again demonstrated a smaller positive association (coefficient, 0.06 mm per week [95% CI, 0.02–0.10]; *P*=0.003). These findings confirm that endometrial thickening is independently associated with ovarian cysts across imaging modalities (Table 5).

**Table 5:** Multivariable linear regression analysis for endometrial thickness

Variable	Ultrasound model	MRI model
Ovarian cyst diagnosis (yes vs. no)	1.56 (0.84–2.26), *P*<0.001	0.81 (0.41–1.21), *P*<0.001
Gestational age (per week)	0.08 (0.04–0.12), *P*<0.001	0.06 (0.02–0.10), *P*=0.003

Models adjusted for gestational age. Ultrasound model: *n* = 46; MRI model: n = 19. *CI*, confidence interval; *MRI*, magnetic resonance imaging

### Diagnostic performance

Receiver operating characteristic (ROC) curve analysis demonstrated excellent performance for endometrial thickness. An ultrasound threshold of 1.9 mm classified all cases correctly, with sensitivity and specificity of 100% and an area under the curve (AUC) of 1.00 (95% CI, 1.00–1.00). The corresponding positive and negative predictive values were also 100%.

In contrast, cyst volume showed only moderate discriminatory ability. A threshold of 11.9 mL yielded a sensitivity of 90% and specificity of 78%, with an AUC of 0.82 (95% CI, 0.71–0.93). At this threshold, the positive predictive value was 87.7% and the negative predictive value was 81.6%.

## Discussion

This study identifies fetal endometrial thickness as a novel and reliable marker for the prenatal diagnosis of ovarian cysts. We found that endometrial thickness, measured on both ultrasound and MRI, was significantly greater in fetuses with ovarian cysts compared with those with non-ovarian cysts, and that this association persisted after adjusting for gestational age. Importantly, endometrial thickness achieved perfect diagnostic accuracy in our cohort, outperforming cyst volume and traditional morphologic assessment.

The accurate prenatal diagnosis of ovarian cysts remains challenging because morphologic features frequently overlap with those of non-ovarian lesions, particularly when cysts are complicated or hemorrhagic. In such cases, reliance on morphologic descriptors alone may lead to diagnostic uncertainty. Our findings demonstrate that incorporating endometrial thickness into prenatal imaging protocols provides an objective and quantifiable marker that enhances diagnostic confidence. Early and accurate recognition of ovarian cysts has important clinical implications, as nearly one-third of cases in our study were complicated by torsion, which can result in ovarian loss and compromise future fertility.

The biological plausibility of endometrial thickness as a diagnostic marker is supported by established developmental physiology. Estrogen receptors are expressed in the fetal endometrium by the late second and third trimesters, and histologic studies confirm responsiveness to transplacental maternal and placental estrogen. A thickened endometrium may therefore serve as a visible indicator of ovarian cysts stimulated by transplacental hormonal exposure. To our knowledge, this is the first study to systematically evaluate endometrial thickness as a diagnostic discriminator, and the accuracy achieved (area under the curve=1.00) represents an important advance over existing approaches that rely solely on morphologic assessment.

Although cyst volume was significantly greater in fetuses with ovarian cysts, this association was no longer significant after adjusting for gestational age. This suggests that gestational age and variability in cyst development may explain much of the observed difference between groups. The moderate diagnostic accuracy of volume measurements (AUC=0.82) supports its use as a complementary rather than primary diagnostic marker. Clinically, larger cysts remain important to monitor given their higher risk of torsion and need for intervention, but cyst volume alone is not sufficient to reliably distinguish ovarian from non-ovarian lesions.

Strengths of this study include the use of both ultrasound and MRI for prenatal assessment, blinded independent review by radiologists with varying levels of experience, and postnatal confirmation of diagnosis through imaging or pathology when available. Together, these factors enhance the robustness and reproducibility of our findings. Limitations include the retrospective single-center design, relatively modest number of MRI cases, lack of formal interobserver reproducibility assessment, and incomplete availability of surgical pathology given that many cysts resolved spontaneously. Future studies should aim to validate these findings prospectively across multiple centers, evaluate reproducibility, and develop standardized protocols for measurement. Longitudinal studies examining the evolution of endometrial thickness across gestations and its correlation with maternal hormone levels may also provide further insights into the underlying physiologic mechanisms.

## Conclusion

Fetal endometrial thickness is a robust imaging marker for the prenatal diagnosis of ovarian cysts. Incorporating this parameter into routine prenatal ultrasound protocols can substantially improve diagnostic accuracy, complement morphologic assessment and cyst volume, and ultimately enhance prenatal counseling and perinatal management.

## Data Availability

The datasets generated and/or analyzed during the current study are not publicly available due to patient privacy but are available from the corresponding author on reasonable request.
